# Unsteady Flow Loss Mechanism and Aerodynamic Improvement of Two-Stage Turbine under Pulsating Conditions

**DOI:** 10.3390/e21100985

**Published:** 2019-10-10

**Authors:** Rongchao Zhao, Weihua Li, Weilin Zhuge, Yangjun Zhang

**Affiliations:** 1 School of Mechanical and Automotive Engineering, South China University of Technology, Guangzhou 510641, China; 2 State Key Laboratory of Automotive Safety and Energy, Tsinghua University, Beijing 100084, China

**Keywords:** two-stage turbine, pulsating flow, flow loss analysis, flow control, computational fluid dynamics

## Abstract

The developments of two-stage turbocharging and turbocompounding promote the application of the two-stage turbine system in internal combustion engines. Since the turbine suffers from the pulsating exhaust, the performance deteriorates significantly from steady conditions. In the paper, the pulsating flow losses in the two-stage turbine are analyzed and a control method is proposed to improve the turbine performance. ANSYS CFX, which is a commercial software for computational fluid dynamic, is applied to resolve the three-dimensional unsteady flow problem. The accuracy of the simulation method is verified by the experimental data from each turbine. Firstly, the impacts of pulse amplitudes on transient loss of each component of the two-stage turbine are studied. Then flow field analysis is carried out to understand details of the unsteady flows. It is found that the variation of incidence angle at the low-pressure turbine (LPT) rotor tip is significantly larger than that at rotor hub, which causes severe flow loss near leading edge. As a result, the LPT performance drops down significantly. To improve the LPT performance, the blade shape at tip is modified. The aerodynamic performances of turbines with three different shapes under high- and low-load pulsating flow conditions are evaluated. It is found that increased inlet blade angle and medium thickness achieves good aerodynamic performance. The rotor averaged efficiency is improved by 2.27% under high-load pulsating condition.

## 1. Introduction

Turbocharging is increasingly important in reducing the fuel consumption and emissions of internal combustion engines [1,2]. However, due to the engine pulsating exhaust, the turbine operates at highly unsteady conditions. It is widely recognized that the turbine performance under pulsating flows deviates from the steady conditions, resulting in large flow loss. In the past decades, a number of studies have been carried out to understand and model the turbine’s unsteady characteristics [3]. However, it is still difficult to improve the turbine efficiency under pulsating flows. Moreover, the previous studies are mainly focused on single stage turbines instead of multi-stage turbine systems. 

The impacts on different kinds of single stage turbine including double-entry turbine [4], waste-gated turbine [5,6], nozzled turbine [7] and variable geometry turbine [8] were studied. The characteristics of radial and mixed-flow turbines under pulsating flow conditions were disclosed in the investigations of Baines [9,10]. Under pulsating flow condition, the curve of mass flow rate versus expansion ratio is a hysteresis loop, which moves around the steady state performance curve. Due to the large difference between the pulsating flow and steady flow conditions, turbine efficiency drops drastically. Unsteady turbine performance is significantly influenced by the pulse amplitude and frequency. Average turbine efficiency improves with higher pulse frequency [11,12]. However, the pulse amplitude shows a disadvantageous impact on turbine performance. As the amplitude increases, the area enclosed by the hysteresis becomes larger, leading to larger turbine flow loss. Mingyang [13] proposed a method to comprehensively evaluate the influence of frequency and amplitude on a mixed-flow rotor. The study showed that the product of Strouhal number (*St*) and the pulse magnitude (*M*), St⋅M can effectively reflect turbine unsteadiness. 

Based on the understanding of the unsteady turbine characteristics, a number of models were developed to capture the unsteady effects. These models are helpful in turbine preliminary design and performance evaluation [14,15,16,17,18,19,20,21]. Also, the performance of engines matched with different turbines can be predicted before the experiment. In Chen’s model [14], the volute of the mixed-flow turbine is simulated as a tapered duct with one-dimensional unsteady flow, while the flow in the rotor is treated as quasi-steady. Empirical flow loss models were adopted to predict the flow loss in the rotor. The predicted fluctuating component of turbine power is in good agreement with the measurements. Serrano [15] and Galindo [16] developed a model of radial turbines appropriate to be used in internal combustion engine simulation. In the model, the turbine is represented by two ideal nozzles, which reproduce the pressure drops across the stator and the rotor. The prediction results agree well with the on-engine test data and computational fluid dynamics (CFD) results. Cao [17] established a low-order model for predicting unsteady performance of radial turbocharger turbines. The model combines an unsteady quasi-three-dimensional computational fluid dynamics method with multiple one-dimensional meanline impeller solvers. It can both consider the wave dynamics in the volute and the rotor inlet circumferential nonuniformity, thus improve the prediction accuracy. 

The abovementioned research disclosed the unsteady characteristic of turbine pulsating flows and proposed modelling methods, while the flow details inside the turbine were not revealed. To explore the flow field inside the turbine, Karamanis firstly measured the flows at the inlet and exit of a mixed-flow turbine under pulsating flow conditions by using a laser Doppler velocity system [18]. The experiment showed that the changing range of the inlet incidence angle during a pulse period is −83 to 52 degrees. The exit deviation angle also changed greatly from −17 to 26 degrees. As a result, the turbine cycle-averaged efficiency dropped 4%–25%. Subsequently, Palfreyman [19] carried out a 3-D unsteady computational study on the same mixed-flow turbine. It showed that the flow field within the turbine stage was highly disturbed and influenced primarily by the pulsating inlet condition. Padzillah [20] investigated the influence of pulsating flow frequencies on the flow angle distributions of a mixed-flow turbine. It was shown that the flow angle fluctuation under unsteady conditions was up to 3 times its steady state equivalence. Peter [21] quantitatively discussed the flow loss breakdowns within the turbine under pulsating flows based on 3-dimensional computational fluid dynamics. The detailed analysis on turbine internal flows provided useful information for better aerodynamics design. 

Control methods were proposed to improve turbine aerodynamics performance [22,23,24,25,26,27,28]. As for passive control methods, Zhang [22] pointed out that rotor leading-edge sweep can reduce the flow field sensitivity to the oscillating inlet flow conditions, and thus improve the turbine average efficiency by 2 percent under pulsating flow conditions. Fredriksson [23] also confirmed that the mixed-flow forward-swept turbine could extract more exhaust energy under pulsating conditions. Active control methods can be found in the investigations of Apostolos [24] and Srithar [25], in which the concept of active control turbocharger (ACT) was proposed. A restrictor was added at the inlet of the rotor to control the flows. The opening degree of the restrictor can be varied periodically according to the pulse. Experimental data showed 2.5%–7.5% more energy was recovered by the turbine when using this method [24]. However, due to the complexity and high cost for the active control systems, they have not yet been applied in automotive engines. 

The above-mentioned research is mainly concerned with single-stage turbines. However, as the development of turbo-compound [26,27] and two-stage turbocharging technologies [28] has progressed, two-stage turbine systems have been widely applied in automotive engines. Zhao [29] reported the characteristics of a two-stage turbine system under steady and pulsating flow conditions. It was found that the load split between the high-pressure turbine (HPT) and low-pressure turbine (LPT) was changed under pulsating condition. More exhaust energy was split to the LPT as the pulse amplitude increased. In addition, the LPT performance deteriorated more significantly under pulsating condition, when compared with the HPT.

In the paper, the flow loss mechanism of the two-stage turbine system under pulsating flow condition will be explored and turbine aerodynamic performance will be improved based on the analysis. Firstly, three-dimensional unsteady CFD model for the two-stage turbine is established and the method is verified. Then, the energy loss in each component of the two-stage turbine during the pulse period will be discussed. The flow details in the rotor during the pulse will be presented. It is found that the large variation of incidence angle near the rotor tip is a main reason leading to large loss. Finally, the impacts of LPT rotor tip design on the unsteady flows and turbine performance will be reported.

## 2. Numerical Method

**Figure 1:**
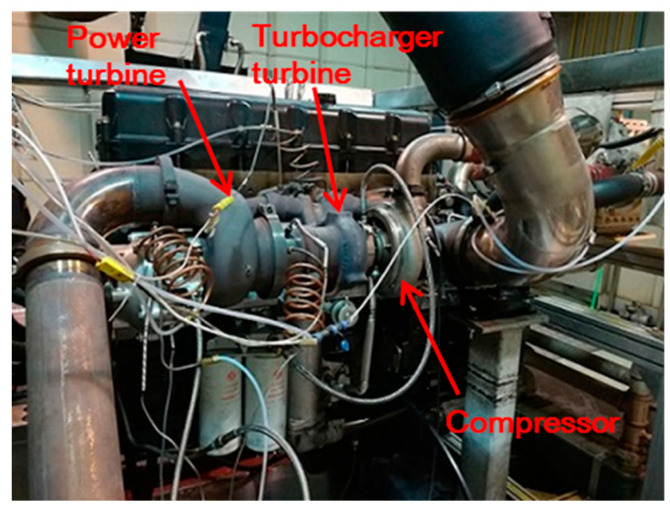
The two-stage turbine installed on engine.

**Figure 2:**
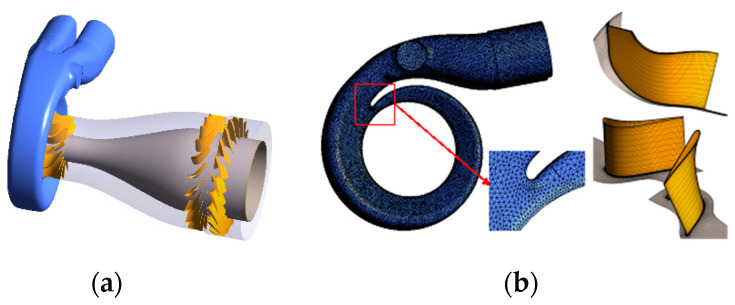
Calculation domain of the two-stage turbine: (**a**) the geometry of two turbine stages; (**b**) mesh of the two turbine stages.

The two-stage turbine is applied in a turbo-compound engine for waste heat recovery [30], as shown in Figure 1. The configuration of the two-stage turbine is shown in Figure 2a. The HPT is a radial-inflow turbine and the LPT is an axial-flow turbine. The two turbine stages are connected via a short intermediate annular duct. The HPT acts as a turbocharger turbine, which extracts the energy from the exhaust and uses it for boosting. The LPT acts as a power turbine for waste heat recovery. The recovered energy is transferred to the engine crankshaft to increase the engine power output and reduce fuel consumption. 

### 2.1. CFD Set-Up

The main geometry parameters of the two-stage turbine are given in Table 1 and Table 2, respectively. The HPT rotor had 12 blades and the LPT had 19 vanes and 20 blades. The diameters of the HPT and LPT rotors were 84 mm and 132 mm, respectively. Both of the rotor tip gaps were 0.5 mm.

The computational fluid dynamics commercial software ANSYS CFX was applied to solve the unsteady flows in the two-stage turbine. The meshes of the volute, rotors and stator are shown in Figure 2b. The quality of the mesh has important effects on the accuracy of the simulation [31]. In this paper, the unstructured grid in the HPT volute was created by using the module MESHING in ANSYS. The mesh quality of HPT volute is shown in Table 3. The structured grids in the rotors and stator passage were created in the module TURBOGRID. High quality of structured grids was obtained with the method, as shown in Table 4. Finally, the four calculation domains were assembled in CFX-PRE. There were a total of 2.03 million grid elements in the calculation activities, with 1.1 million in volute, 0.24 million in HPT rotor, 0.33 million in LPT stator and 0.36 million in LPT rotor.

To reduce the calculation time, only a single passage was modelled in each stage. Rotational periodicity of each stage was set up in the calculation. The periodicities in HPT rotor, LPT stator and rotor were 12, 19 and 20, respectively. In such a way, the flows in other passages were assumed to be the same as the calculated passage in one stage. This is a generally used method to reduce the requirement for the computational resources [32,33]. The mixing plane method was adopted at the three rotor/stator interfaces. Single passage combined with mixing plane method was reported in literature [32] for two-stage flows under pulsating conditions. In the mixing plane method, the rotor/stator interaction is done by exchanging circumferentially averaged flow quantities. Physically, it means that the wake and separation generated in the upstream component are mixed circumferentially before entering the downstream component. Therefore, the velocity components and pressure are uniform in the circumferential direction at the inlet of the downstream component. Using this method, whole passage calculation can be replaced by single passage modeling, reducing the calculation time significantly. However, it can only be used when the circumferential distortion is not significant. Considering that the primary problem in the current study is the unsteadiness caused by the pulse, the rotor/stator unsteady effects and circumferential distortion were neglected in the paper. Therefore, mixing plane is a reasonable method for predicting two-stage flows under pulsating conditions. 

The time step of the simulation was set as 1% of the pulse period. This means that there are 100 calculation points during one pulse period, which is enough to capture the variation of the pulse effect. Cyclic convergence could be obtained after three pulse cycles in the current study. Each unsteady case calculation takes approximately 20 hours to complete, based on 12 CPU cores working in parallel.

It should be pointed out that the time step was not determined according to the rotor rotating speed in the current study, since the unsteady interactions between rotor and stator were not considered. If the rotor/stator unsteady interactions are considered, the time step has to be very small. For instance, there are 12 blades in the HPT rotor. If 20 time steps are chosen for each blade passing, then there are 360 time steps in one revolution. The unsteady effects caused by the pulse also need to be considered. However, the time scale for the pulse period is significantly larger than that of rotor rotating period. When the rotating speed is 84,100 rpm and the pulse frequency is 60 Hz, the rotor rotates 23.36 revolutions during one pulse period. Suppose cyclic convergence was obtained after three pulse cycles, the rotor will have rotated approximately 70 revolutions. As a result, at least 25,200 time steps are required for each case of calculation. It would take at least 70 days to complete one calculation case. This extremely long time cannot be endured in the current work. Therefore, the time step in the current study was decided according to the pulse period, which is the main concern of the work. The impacts of time-step size on the calculation result were reported in a previous study carried out by the authors [32]. The mesh independency study is also presented in the reference [32].

A two-equation k-epsilon turbulence model with scalable wall function was used in the simulation. This turbulence model was successfully applied in some previous studies on unsteady flows in turbomachinery [21,34]. 

**Figure 3:**
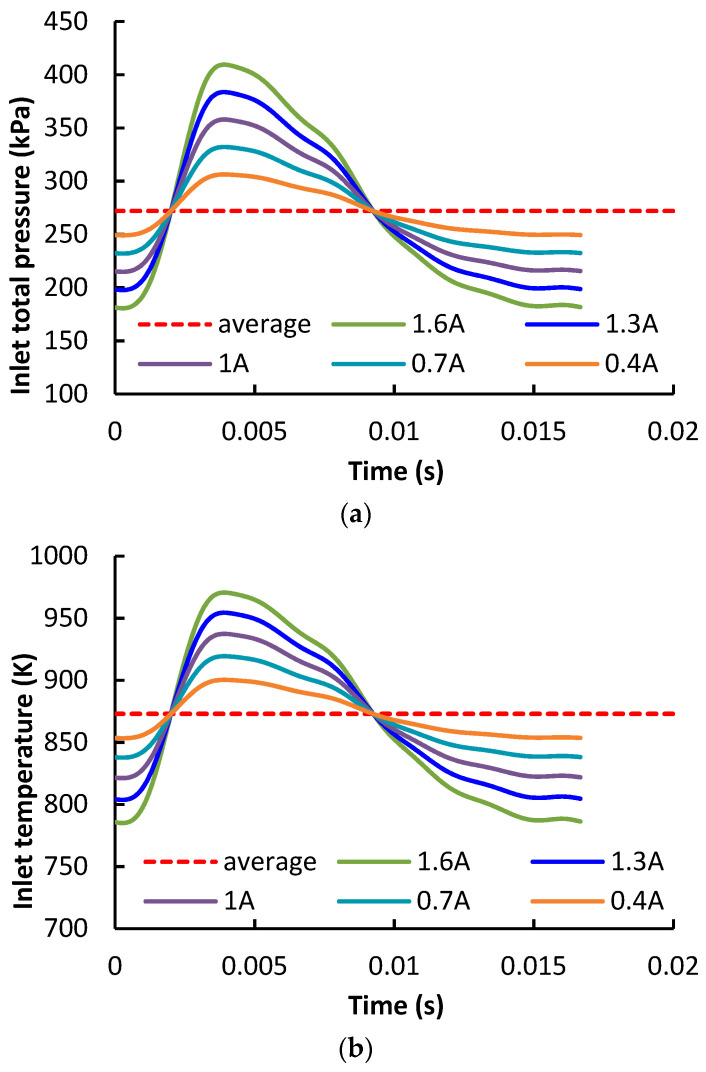
Total (**a**) pressure and (**b**) temperature profiles imposed at the inlet of the calculation domain; (**a**) total pressure; (**b**) total temperature.

Since the engine torque point condition (engine 1200 rpm and full-load condition) is an important operation point of the engine, therefore it is important to improve the turbine efficiency under this condition. At torque point, the rotating speeds of the HPT rotor and LPT rotor are 84,100 rpm and −27,200 rpm, respectively, which were measured from the engine test bench. The time-averaged total pressure and temperature at the HPT inlet were 272 kPa and 873 K, respectively. Time-varying inlet total pressure and temperature profiles are imposed at the inlet boundary. In the current study, the impact of pulse amplitude on the flow loss was studied. As shown in Figure 3, the inlet total pressure profiles are the same as the profiles in [29]. The adjusting method is presented in Equation (1). It ensures that the averaged pressure is kept the same when varying the pulse amplitude. The coefficients φ were set as 0.4, 0.7, 1.0, 1.3 and 1.6 in the five calculation cases. Static pressure was given at the LPT exit as the outlet boundary condition.

(1) $$p_{inst}=p_{ave}+\varphi A=p_{ave}+\varphi (p_{inst,baseline}-p_{ave})$$

### 2.2. CFD Validation

**Figure 4:**
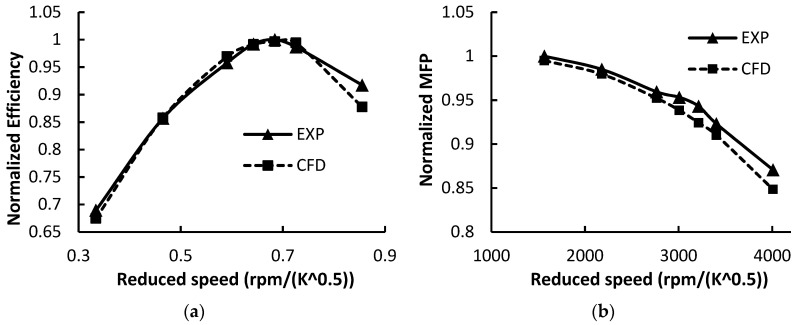
Validation of the computational fluid dynamics (CFD) model under steady condition, HPT expansion ratio $\pi_{h}=2.2$(T-S); (**a**) turbine efficiency; (**b**) mass flow parameter.

The CFD model can well predict the turbine performance under steady and pulsating flow conditions. Since it is difficult to operate the two-stage turbine experiment due to the test bench limitation, the prediction accuracies of the two turbines’ performances are validated separately. Figure 4 presents the comparisons of HPT efficiency and mass flow parameters between the experimental data and CFD results. The definition of blade speed ratio (BSR), mass flow parameter (MFP) and turbine reduced speed are given in Equations (2), (3) and (4), respectively. The normalized efficiency is the ratios of the current efficiency to the peak efficiency in the experiment. The normalized MFP is the ratios of the MFP to the largest MFP in the experiment. BSR is the ratio between the rotor tip velocity and gas spouting velocity. The latter one is related to the turbine inlet total temperature and expansion ratio. For radial-inflow turbine, the optimum BSR is approximately 0.7. As BSR deviates from the optimum value, the turbine isentropic efficiency drops [35].

(2) $$BSR=\frac{r\omega }{\sqrt{2c_{p}T_{t,in}\left[1-{\left(1/\pi \right)}^{(\gamma -1)/\gamma }\right]}}$$

(3) $$MFP=\frac{m\sqrt{T_{t,in}}}{p_{t,in}}$$

(4) $$N=\frac{n}{\sqrt{T_{t,in}}}$$

The turbine experimental performance map is provided by the manufacturer. The curves shown in Figure 4 are obtained under the condition that the expansion ratio (T-S) equals 2.2. The difference of efficiency between the EXP and CFD is small under low and medium blade speed ratio conditions. At high blade speed ratio condition, the largest difference is 4.33%. As for the MFP prediction, the average difference is 1.33%. As for the unsteady calculation, the method applied in the current study is the same as that in the previous study [29]. Therefore, the simulation method in the current study is trustworthy.

**Figure 5:**
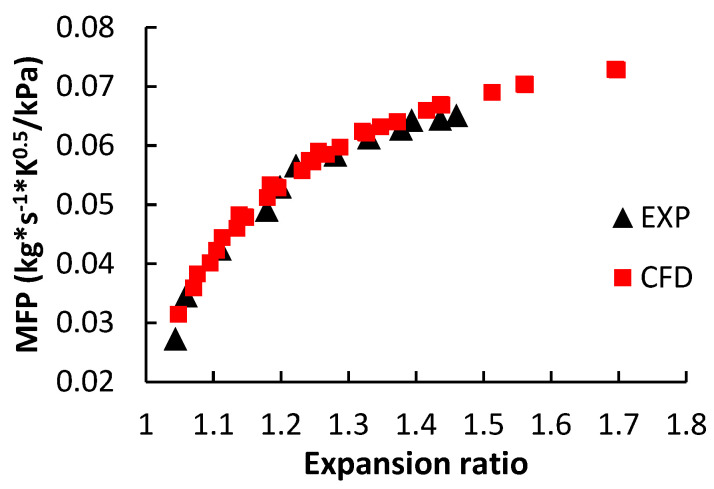
Comparisons between the experimental and CFD results of the LPT mass flow parameter.

As for the LPT performance validation, Figure 5 compares the MFP results from experiment and CFD method. The LPT experimental data is measured from the on-engine test bench. The engine air mass flow rate, turbine inlet temperature and pressure were measured in the engine performance test. Thus, the curve of MFP versus expansion ratio was obtained. The details on the engine experiment can be seen in the literature [30]. 

## 3. Flow Loss Analysis of Two-Stage Turbine under Pulsating Conditions

**Figure 6:**
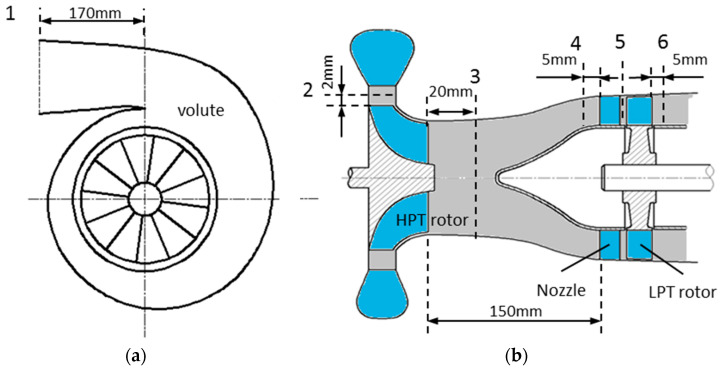
Schematic of the two-stage turbine and the locations for monitor; (**a**) HPT volute; (**b**) Cross-sectional view of the two-stage turbine.

The two-stage turbine flows under five different pulse amplitude conditions were resolved in this study. During the calculation, some variables at each time step were monitored. The monitored variables included instant total pressure and temperature, static pressure and temperature, mass flow rate at the locations shown in Figure 6. In addition, the instant torques of the HPT and LPT rotors were also recorded.

As shown in Figure 6, the locations for monitor included volute inlet, HPT rotor inlet and exit, LPT nozzle inlet, rotor/stator interface, and LPT rotor exit. The distance between the volute inlet and the tongue is approximately 170 mm. Location 2 is 2 mm upstream the HPT rotor leading edge and location 3 is 20 mm downstream the HPT rotor trailing edge at hub. Locations 4 and 6 are 5 mm upstream the nozzle leading edge and 5 mm downstream the LPT rotor trailing edge at hub, respectively. Location 5 is the interface of the rotor/stator, which is approximately 4 mm away from the nozzle trailing edge and LPT rotor leading edge. 

Under ideal condition, the instant isentropic expansion power of the two-stage turbine can be expressed in Equation (5). The actual instant power of the rotors is a function of torque and speed, as shown in Equation (6).

(5) $$P_{s,inst}=m_{1}c_{p}T_{t1}\left[1-{\left(p_{6}/p_{t1}\right)}^{(\gamma -1)/\gamma }\right]$$

(6) $$P_{a,inst}=\tau \omega$$

The cycle-averaged isentropic power and actual power of the two-stage turbine system are defined as Equations (7) and (8), respectively. Therefore, averaged efficiency of the two-stage turbine is obtained as Equation (9).

(7) $$P_{s,ave}=\frac{\int_{0}^{T}P_{i,inst}dt}{T}$$

(8) $$P_{a,ave}=\frac{\int_{0}^{T}P_{a,inst}dt}{T}$$

(9) $$\eta_{ave}=\frac{P_{a,ave}}{P_{i,ave}}$$

**Figure 7:**
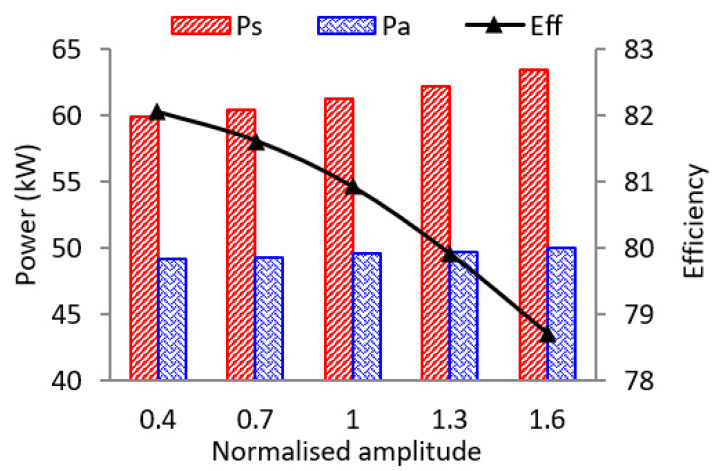
The impacts of pulse amplitude on turbine theoretical power, actual power and isentropic efficiency.

Figure 7 presents the impacts of pulse amplitude on the two-stage turbine averaged isentropic power, actual power and isentropic efficiency. It is shown that even though the averaged inlet total pressure is kept the same in the five cases, the theoretical isentropic expansion power increases as the pulse amplitude increases. As the amplitude increases from 0.4 A to 1.6 A, the theoretical power increases by 6%. In addition, the actual power also increases 1.7%. However, the isentropic efficiency drops from 82.06% to 78.72%. It can be seen that even though stronger pulse will lead to lower turbine efficiency, it still produces more actual power. Therefore, the time-averaged inlet pressure cannot decide the available energy in the exhaust. If the pulse shape becomes flatter after it transfers in the pipe system, the available energy also becomes less. Thus, it is important to preserve the pulse amplitude when it is transferred in the exhaust system. 

**Figure 8:**
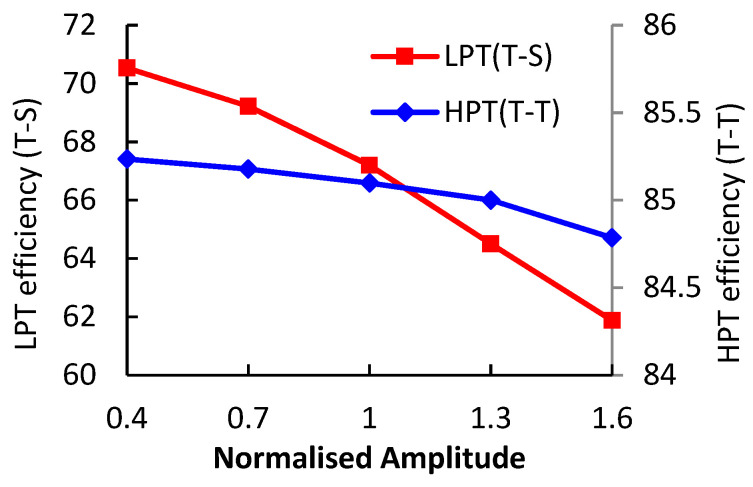
The impacts of pulse amplitude on turbine average isentropic efficiency.

The impacts of the pulse amplitude on average isentropic efficiency of each turbine stage are illustrated in Figure 8. The efficiency drop of LPT is evidently larger than that of HPT. When the pulse amplitude increases from 0.4 A to 1.6 A, the HPT efficiency drops by 0.45% while the LPT efficiency drops by 8.66%. To understand the flow loss in the two-stage turbine system deeply, the instant flow loss in each component was analyzed.

The flow losses in five components, including volute, HPT rotor, intermediate duct, LPT stator and rotor, were calculated by Equations (10), (11), (12), (13) and (14), respectively. Suppose that the static pressure at location 6 is kept constant, the maximum expansion power produced by the fluid at location 1 (*P*_s,1–6_) is obtained by the isentropic process from point 1 to point 6. Similarly, the maximum expansion power produced by the fluid at location 2 is denoted as *P*_s,2–6_. Thus, the power loss in HPT volute can be expressed as Equation (10). In the calculation of the loss in rotor component, the actual power output of the rotor needs to be excluded, as shown in Equations (11) and (14). Since there is a phase lag between the upstream and downstream components, the direct subtraction may result in a negative value of the power loss. Obviously, this is physically incorrect. Therefore, the profiles of the power versus time of the downstream component are advanced to be in phase with that of the upstream component. This manipulation method will not affect the cycle-averaged power since the exit pressure *p*_6_ is kept constant.

(10) $$P_{loss,1-2}=P_{s,1-6}-P_{s,2-6}$$

(11) $$P_{loss,2-3}=P_{s,2-6}-P_{s,3-6}-P_{a,hpt}$$

(12) $$P_{loss,3-4}=P_{s,3-6}-P_{s,4-6}$$

(13) $$P_{loss,4-5}=P_{s,4-6}-P_{s,5-6}$$

(14) $$P_{loss,5-6}=P_{s,5-6}-P_{a,hpt}$$

**Figure 9:**
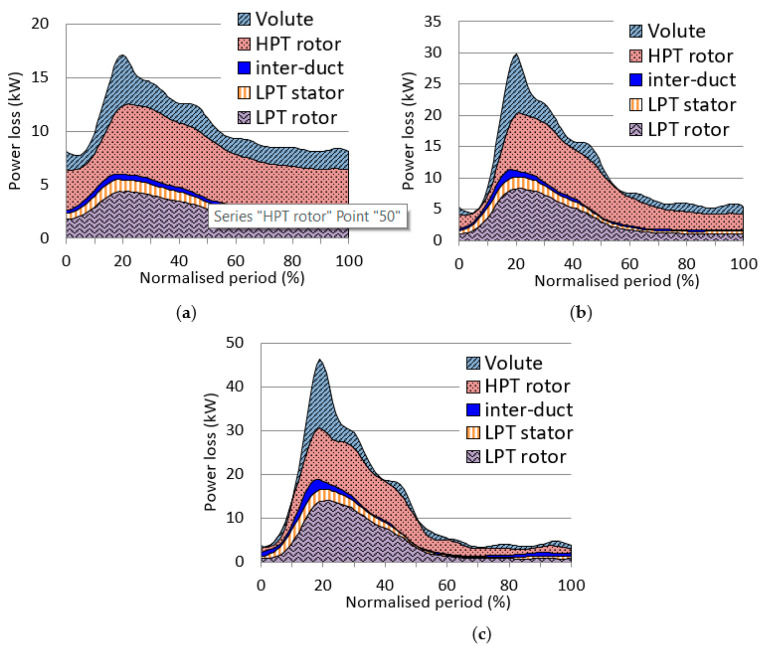
The flow losses in each component during a pulse period; (**a**) the pulse amplitude is 0.4 A; (**b**) the pulse amplitude is 1.0 A; (**c**) the pulse amplitude is 1.6 A.

The power losses in each component as a function of time are shown in Figure 9. The peak of the loss appears at 20% of the pulse period, corresponding to the peak pressure. It should be noticed that the peak losses in the LPT components actually appears at approximately 28% of the pulse period due to phase lag. The current figure has been manipulated to make them in phase. As shown in Figure 9a, the power losses mainly occur in the HPT rotor and LPT rotor. The power losses are significant in the whole pulse period. The loss in volute is also significant. In contrast, the losses in inter-duct and LPT stator are nearly neglected. 

As the pulse amplitude increase, the power losses centralize more at 20% pulse period. When the pulse amplitude is 1.6 A, more than 80% of the power loss occurs in 10–60% of the pulse period. The peak instant power loss also increases significantly. As shown in Figure 9, the peak power losses under the three pulse amplitude conditions are approximately 17, 30 and 46kW, respectively.

**Figure 10:**
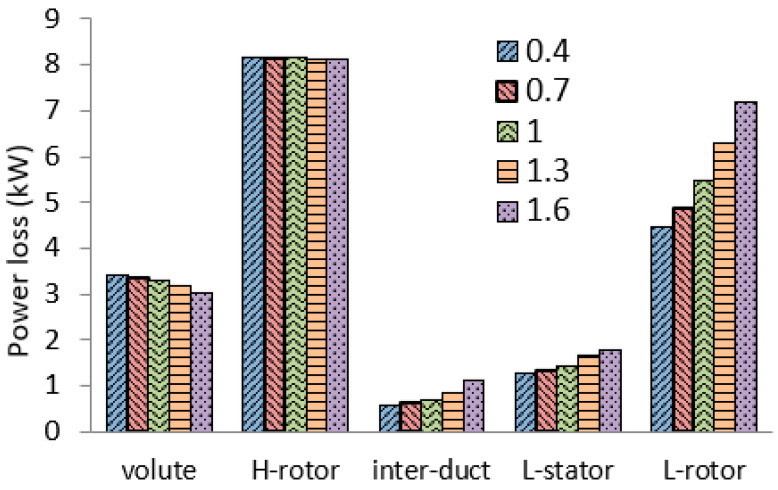
The impacts of the pulse amplitude on the average power loss in each component.

The impacts of the pulse amplitude on the cycle-averaged power loss in each component are shown in Figure 10. As the pulse amplitude increases, the power losses in volute and HPT rotor decrease slightly. It is an interesting phenomenon and seems to be unresonable. Actually, the pulse amplitude will affect the power split between the HPT and LPT, which was reported in a previous study [29]. As the pulse amplitude increases, more power will be distributed to the LPT. Therefore, the power split to the HPT will be less. As a result, the power loss will become less. However, the HPT efficiency will still decrease. In contrast, the power losses in the LPT components increase significantly as the pulse amplitude increases. Especially in LHT rotor, the power loss increases by 60% when the pulse amplitude increases from 0.4 A to 1.6 A. 

**Figure 11:**
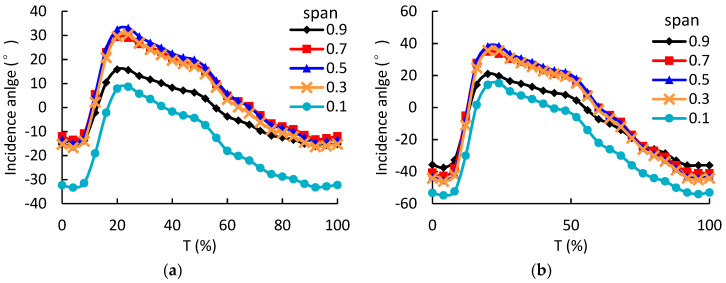
The circumferential-averaged incidence angle at different span of the HPT rotor as a function of time; (**a**) HPT rotor with 1.0 A pulse amplitude; (**b**) HPT rotor with 1.6 A pulse amplitude.

To understand the flows in the rotors deeply, Figure 11 presents the HPT rotor incidence angle at different spans under 1.0 and 1.6 A pulse amplitude conditions. It is shown that the incidence angles as a function of time are quite uniform at 0.3, 0.5 and 0.7 spans. The incidence angles at 0.1 and 0.9 spans are smaller than those at other spans. This is mainly due to the viscous effects on the sidewall, which lead to lower inlet velocity. Eventually, the incidence angle will become smaller. 

**Figure 12:**
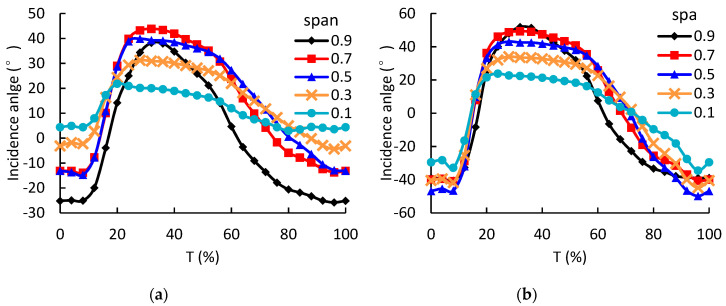
The circumferential-averaged incidence angle at different span of the LPT rotor as a function of time; (**a**) LPT rotor with 1.0 A pulse amplitude; (**b**) LPT rotor with 1.6 A pulse amplitude.

The variation of incidence angles of LPT rotor are quite different at different spans, as shown in Figure 12. At 0.1 span, the varying range of the incidence angle during a pulse period is the smallest. Along the spanwise direction, the vaying range of the incidence angle increases. Under 1.6 A pulse amplitude condition, the angle varying ranges at 0.1 and 0.9 spans are 58.3 and 92.6 degrees, respectively. Obviously, the larger incidence angle variation will lead to lager flow loss in the rotor passage. Therefore, the energy loss at the blade tip will be significnatly higher than that at the blade hub when subjected to pulsating flows.

**Figure 13:**
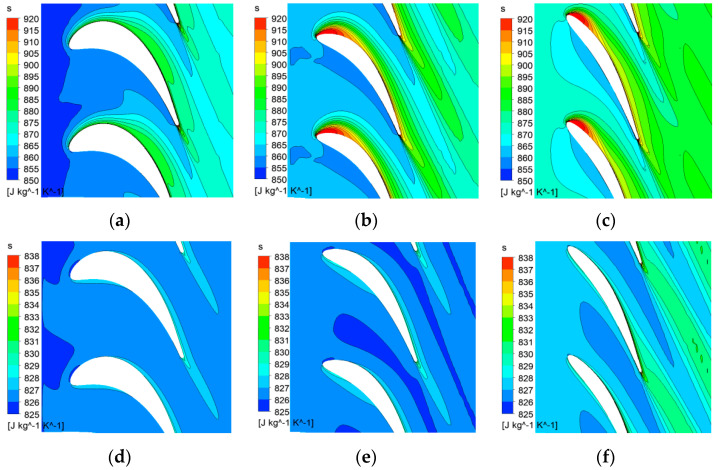
The entropy contour at 20%, 50% and 80% span of the LPT rotor at different times when the pulse amplitude is 1.6 A; (**a**)peak time, 20% span; (**b**)peak time, 50% span; (**c**)peak time, 80% span; (**d**)100% T, 20% span; (**e**)100% T, 50% span; (**f**)100% T, 80% span.

Since the LPT performance is more sensitive to the pulsating flow conditions when compared with HPT, the following discussion will be mainly focused on the LPT flow. Figure 13 presents the entropy contour at 20%, 50% and 80% span of the LPT rotor when the pulse amplitude is 1.6 A. At peak time condition, the incidence angle at tip is significantly larger than that at hub. As a result, high entropy generation was generated at leading edge of the suction side due to large incidence angle at 50% and 80% span of the rotor. At 20% span location, the entropy along the suction side is much lower than those at 50% and 80% span.

At 100% pulse period, the entropy near rotor tip is also larger than near hub. As shown in Figure 13d–f, high entropy values appear at the leading edge of the pressure side due to negative incidence angle. Since the velocity at 100% T is much lower, the entropy loss is significantly lower than that at peak time condition. However, it can still be observed that the entropy loss near tip is a lot larger than that near hub. 

From the above analysis, it can be concluded that the flow at LPT rotor tip is more sensitive to the pulsating flow, resulting in large flow loss near tip. Therefore, it is important to improve the aerodynamic performance near rotor tip under pulsating flow conditions.

## 4. Aerodynamic Performance Improvement

**Figure 14:**
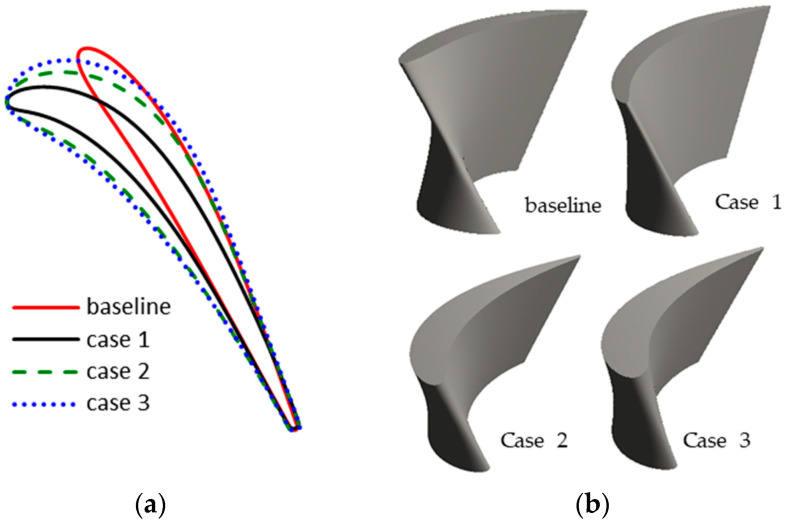
Comparisons of different blade designs; (**a**) blade profiles at blade tip; (**b**) 3D view of the rotor blade.

From the above analysis, the large variation of inlet incidence angle near the blade tip leads to significant flow loss under pulsating flow conditions. To alleviate the flow separation near the tip region, it is necessary to adjust the inlet blade angle distribution. Since the most exhaust energy is contained at the peak during a pulse period, the rotor blade angle should be redesigned to extract more energy at peak. In addition, blades with different thickness are designed in order to explore its impacts on the unsteady turbine performance. Thus, three different blade shapes at tip are designed, as shown in Figure 14, in comparison with the baseline blade tip. 

In case 1, the blade inlet angle is increased to adapt to the large flow incidence angle, while the thickness is kept the same. Therefore, the turbine aerodynamic performance at large incidence angle condition can be improved with case 1. However, the flow loss at negative incidence angle conditions may be increased. To reduce the aerodynamic sensitivity to the inlet incidence angle, another two cases were designed with different thickness. The blade angles in cases 2 and 3 are the same as that in case 1. However, the thicknesses are different. The blade in case 3 is the thickest one and the blade in case 1 is the thinnest. With a thicker blade, the curvature radius at the leading edge increase greatly. It may obtain better aerodynamic performance under varying incidence angle conditions. 

It can be easily seen that the curvature of the leading edge becomes significantly smaller with a larger thickness. The larger thickness will lead to larger mass and momentum of the rotor. Considering that the operation temperature and rotation speed of the power turbine is relatively low, the increased centrifugal force caused by the larger mass will not excess the stress limit. The aerodynamic performances of the three rotors under high- and low-load pulsating flow conditions were evaluated.

**Figure 15:**
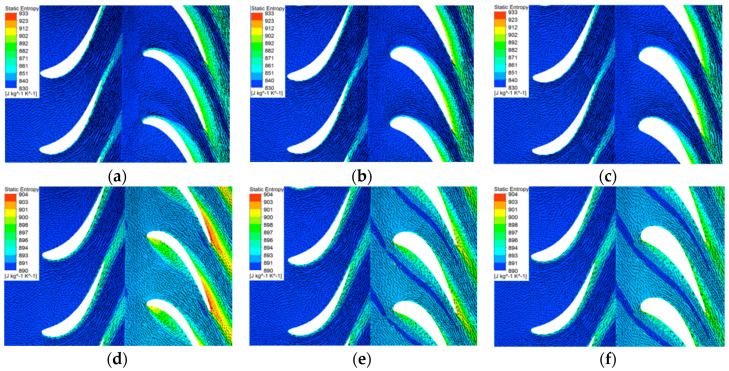
The entropy contour and velocity plot at 80% span of the rotor; (**a**) case 1, BSR = 0.35; (**b**) case 2, BSR = 0.35; (**c**) case 3, BSR = 0.35; (**d**) case 1, BSR = 0.95; (**e**) case 2, BSR = 0.95; (**f**) case 3, BSR = 0.95.

The rotor performance under two extreme conditions (BSR = 0.35 and BSR = 0.95) were analyzed. Figure 15 presents the entropy contour and velocity plot at 80% span of the rotor under high-load and low-load conditions in three cases. In case 1 when the BSR equals 0.35, the inlet blade angle is adapted to the large inlet flow angle. Consequently, the flow separation on the suction side is significantly suppressed. However, the aerodynamic performance of the blade in case 1 deteriorates when BSR equals to 0.95. The incidence angle becomes negative under this condition. As a result, large flow separations occur on the pressure side of the blade near the leading edge. Moreover, severe entropy loss also appears near the trailing edge on the suction side. 

In cases 2 and 3, the blade thickness is larger. Although the incidence angle is still large when BSR = 0.35, large flow separations are not observed in both cases 2 and 3. The entropy loss in the rotor passage is decreased significantly when compared with the baseline rotor. Under low-load condition when BSR = 0.95, the incidence angle becomes negative, as shown. However, due to smaller curvature in the leading edge, the flow separation on the pressure side becomes weaker when compared with case 1. The flow separation becomes smaller when the rotor has thicker blades. Thus, a thicker blade lowers the sensitivity to the changing of incidence angle and suppresses flow separation. 

**Figure 16:**
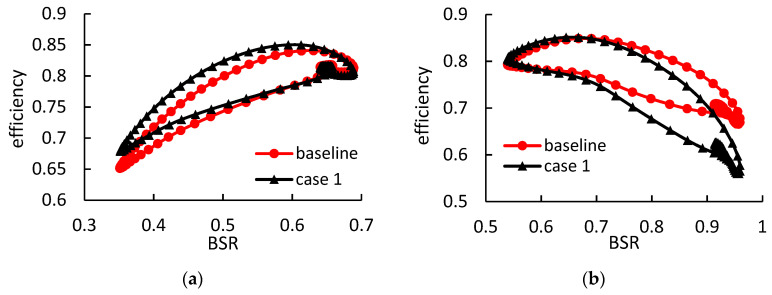
The rotor instantaneous isentropic efficiency during a pulse period in case 1: (**a**) under high-load pulsating flow; (**b**) under low-load pulsating flow.

The rotor instantaneous isentropic efficiency during a pulse period in case 1 is shown in Figure 16. The definition of the rotor instantaneous efficiency is shown in Equation (15).

(15) $$\eta_{inst}=\frac{\tau \omega }{{\dot{m}}_{5}c_{p}T_{t5}\left[1-{\left(p_{6}/p_{t5}\right)}^{\frac{\gamma -1}{\gamma }}\right]}$$

It is shown that the rotor efficiency in case 1 is remarkably improved under low BSR conditions. The maximum improvement of the instantaneous efficiency is up to 3%. However, the rotor efficiency in case 1 decreases rapidly as the BSR increases. The maximum reduction of the instantaneous efficiency is up to 10.5% when the BSR is approximately 0.96. Therefore, it can hardly both improve the performances under high- and low-load conditions by merely adjusting the inlet blade angle. Although the rotor efficiency improves greatly under high-load condition, the performance under low-load condition deteriorates significantly. 

**Figure 17:**
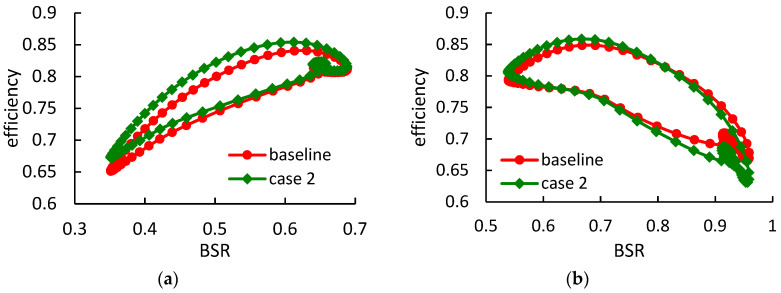
The rotor instantaneous isentropic efficiency during a pulse period in case 2: (**a**) under high-load pulsating flow; (**b**) under low-load pulsating flow.

**Figure 18:**
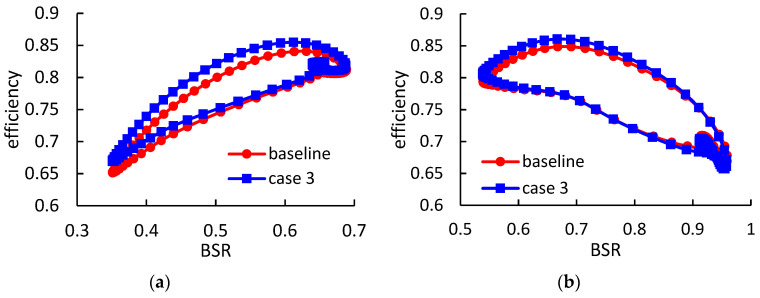
The rotor instantaneous isentropic efficiency during a pulse period in case 3: (**a**) under high-load pulsating flow; (**b**) under low-load pulsating flow.

Figure 17 and Figure 18 show the rotor instantaneous efficiency during a pulse period in case 2 and case 3, respectively. Under high-load condition, the rotor with thicker blades obtains relatively smaller efficiency improvements, when compared with case 1. The maximum instantaneous efficiency improvements in case 2 and case 3, are 2.5% and 2.2% respectively. However, under low-load condition, the thicker blades obtain higher efficiency when compared with case 1. 

**Figure 19:**
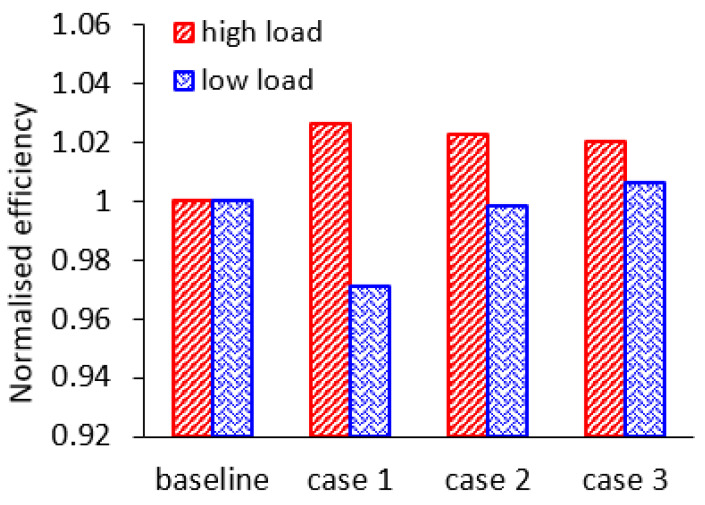
The cycle-averaged rotor efficiency in all cases.

The cycle-averaged efficiencies of the three cases and the baseline under high-load and low-load condition are compared in Figure 19. Compared with others, case 1 obtains the highest averaged efficiency under high-load pulsating condition. The cycle-averaged rotor efficiency in case 1 under high-load condition is improved by 2.63%. However, the rotor efficiency in case 1 under low-load condition is the lowest, which is 2.9% lower than the baseline.

The average efficiency improvements in case 2 and case 3 under high-load conditions are 2.27% and 2.02%, respectively, when compared with the baseline. Furthermore, the average efficiency in cases 2 and 3 under low-load conditions are equal to or slightly higher than the baseline. 

## 5. Conclusions

As the developments of two-stage turbocharging and turbocompounding progress, the two-stage turbine is increasingly widely applied in internal combustion engines. Due to the periodic opening and closing of the exhaust valves, the two-stage turbine system is subjected to highly pulsating flows, resulting in different behavior. However, a large number of investigations are mainly concerned with single stage turbines instead of multi-stage turbines. In this paper, the flow losses inside the two-stage turbine under pulsating conditions are investigated based on three-dimensional unsteady CFD method. The two-stage turbine is composed of a radial-flow high-pressure stage and an axial-flow low-pressure stage. It is found that the low-pressure stage efficiency suffers a lot from the pulsating flow. Thus, a control method is proposed to improve the aerodynamic performance of LPT. The main conclusions are drawn as follows.

(1) As the pulse amplitude increases, the flow loss will more concentrate on the peak pressure time. More than 80% of the flow loss occurs in the 10%–60% of the pulse period when the pulse amplitude is 1.6 A. In addition, it is found that the flow loss in the HPT decreases slightly while that of LPT rotor increases evidently by 60% as the amplitude increases from 0.4 to 1.6 A. Correspondingly, the HPT efficiency only drops by 0.45% while the LPT efficiency drops by 8.66%.

(2) During a pulse period, the varying range of incidence angle at LPT rotor tip is significantly larger than that at LPT rotor hub. Under 1.6 A pulse amplitude condition, the angle varying ranges at 0.1 and 0.9 spans are 58.3 and 92.6 degrees, respectively. The large variation of incidence angle at rotor tip leads to large flow loss near the leading edge. Unlike LPT, the varying patterns of incidence angle of HPT rotor are similar at different spans.

(3) The aerodynamic performances of turbines with three different shapes under high- and low-load pulsating flow conditions are evaluated. The blade inlet angle in case 1 is increased to adapt to the large incidence at peak pressure condition. In cases 2 and 3, the blade inlet angle is the same as that in case 1. However, the thickness of the blades is increased to lower the sensitivity of the leading edge to the varying incidence angle. The efficiency of LPT in case 2 (increased inlet blade angle and medium thickness) is increased by 2.27% under high-load pulsating condition and kept the same under low-load pulsating condition.

## Figures and Tables

**Table 1:** Specifications of the radial-inflow high-pressure turbine (HPT).

Geometrical Feature	Dimension
Volute A/R (mm)	15
Rotor inlet diameter (mm)	84
Rotor inlet height (mm)	9.3
Rotor exit tip diameter (mm)	69
Rotor tip gap (mm)	0.5
Number of blades	12

**Table 2:** Specifications of the axial-flow low-pressure turbine (LPT).

Geometrical Feature	Dimension
Stator tip diameter (mm)	128
Stator hub diameter (mm)	92
Number of vanes in stator	19
Rotor tip diameter (mm)	132
Rotor hub diameter (mm)	90
Rotor tip gap (mm)	0.5
Number of blades in rotor	20

**Table 3:** Mesh quality of the HPT volute.

Maximum Skewness	Minimum Orthogonal	Maximum Aspect Ratio
0.918	0.105	23.59

**Table 4:** Mesh quality of the rotor/stator.

	Minimum Face Angle (°)	Maximum Face Angle (°)	Maximum Element Volume Ratio
HPT rotor	30.47	149.67	6.09
LPT stator	55.14	112.54	3.76
LPT rotor	29.42	151.38	10.19

## Nomenclature

*A*	amplitude	
*c*_p_	specific heat ratio at constant pressure	J/kg K
*m*	mass flow rate	kg/s
*N*	Reduced speed	rpm/K^0.5^
*n*	rotation speed	rpm
*P*	power	kW
*p*	pressure	Pa
*R*_g_	gas constant	J/kg K
*r*	radius	mm
*T*	temperature or pulse period	K or s
*t*	time	s
**Acronyms**		
BSR	blade speed ratio	
CFD	computational fluid dynamics	
EXP	experiment	
HPT	high-pressure turbine	
LPT	low-pressure turbine	
MFP	mass flow parameter	
T-T	total to total	
T-S	total to static	
**Greek symbols**		
γ	adiabatic exponent	
η	efficiency	
π	expansion ratio	
τ	torque	Nm
φ	coefficient	
ω	angular velocity	rad/s
**Subscripts and superscripts**		
1–6	locations in the two-stage turbine	
a	actual	
ave	average	
*in*	inlet	
*inst*	instant	
*s*	isentropic	
*t*	stagnation condition	
